# Asymptomatic *Plasmodium vivax* infections induce robust IgG responses to multiple blood-stage proteins in a low-transmission region of western Thailand

**DOI:** 10.1186/s12936-017-1826-8

**Published:** 2017-04-28

**Authors:** Rhea J. Longley, Camila T. França, Michael T. White, Chalermpon Kumpitak, Patiwat Sa-angchai, Jakub Gruszczyk, Jessica B. Hostetler, Anjali Yadava, Christopher L. King, Rick M. Fairhurst, Julian C. Rayner, Wai-Hong Tham, Wang Nguitragool, Jetsumon Sattabongkot, Ivo Mueller

**Affiliations:** 1 Walter and Eliza Hall Institute of Medical Research, Melbourne, Australia; 2 Mahidol Vivax Research Unit, Faculty of Tropical Medicine, Mahidol University, Bangkok, Thailand; 3 Department of Medical Biology, University of Melbourne, Melbourne, Australia; 4 Imperial College, London, UK; 5 Department of Tropical Hygiene, Faculty of Tropical Medicine, Mahidol University, Bangkok, Thailand; 6 Malaria Programme, Wellcome Trust Sanger Institute, Wellcome Genome Campus, Hinxton, Cambridge, UK; 7 Laboratory of Malaria and Vector Research, National Institute of Allergy and Infectious Diseases, National Institutes of Health, Bethesda, MD USA; 8 Malaria Vaccine Branch, United States Military Malaria Research Program, Walter Reed Army Institute of Research, Silver Spring, MD USA; 9 Center for Global Health and Diseases, Case Western Reserve University, Cleveland, OH USA; 10 Department of Molecular Tropical Medicine and Genetics, Faculty of Tropical Medicine, Mahidol University, Bangkok, Thailand; 11 ISGlobal, Barcelona Institute for Global Health, Hospital Clínic-Universitat de Barcelona, 08036 Barcelona, Spain; 12 Institut Pasteur, Paris, France

**Keywords:** *Plasmodium vivax*, Malaria, IgG, Antibody, Humoral immunity, Vaccine, Elimination, Asymptomatic, Exposure

## Abstract

**Background:**

Thailand is aiming to eliminate malaria by the year 2024. *Plasmodium vivax* has now become the dominant species causing malaria within the country, and a high proportion of infections are asymptomatic. A better understanding of antibody dynamics to *P. vivax* antigens in a low-transmission setting, where acquired immune responses are poorly characterized, will be pivotal for developing new strategies for elimination, such as improved surveillance methods and vaccines. The objective of this study was to characterize total IgG antibody levels to 11 key *P. vivax* proteins in a village of western Thailand.

**Methods:**

Plasma samples from 546 volunteers enrolled in a cross-sectional survey conducted in 2012 in Kanchanaburi Province were utilized. Total IgG levels to 11 different proteins known or predicted to be involved in reticulocyte binding or invasion (ARP, GAMA, P41, P12, PVX_081550, and five members of the PvRBP family), as well as the leading pre-erythrocytic vaccine candidate (CSP) were measured using a multiplexed bead-based assay. Associations between IgG levels and infection status, age, and spatial location were explored.

**Results:**

Individuals from a low-transmission region of western Thailand reacted to all 11 *P. vivax* recombinant proteins. Significantly greater IgG levels were observed in the presence of a current *P. vivax* infection, despite all infected individuals being asymptomatic. IgG levels were also higher in adults (18 years and older) than in children. For most of the proteins, higher IgG levels were observed in individuals living closer to the Myanmar border and further away from local health services.

**Conclusions:**

Robust IgG responses were observed to most proteins and IgG levels correlated with surrogates of exposure, suggesting these antigens may serve as potential biomarkers of exposure, immunity, or both.

**Electronic supplementary material:**

The online version of this article (doi:10.1186/s12936-017-1826-8) contains supplementary material, which is available to authorized users.

## Background

Thailand is a region of low malaria transmission, with approximately 52,000 (16,000–150,000) cases estimated in 2015 [1]. The country is currently within the ‘control’ phase per the World Health Organization, and the National Malaria Strategy proposes to eliminate malaria by the year 2024 (Department of Disease Control, Ministry of Public Health, Thailand). This is part of a wider goal of a malaria-free Asia–Pacific by the year 2030, declared at the 9th East Asia Summit in 2014 by the Asia–Pacific Leaders Malaria Alliance. As malaria transmission has decreased in Thailand, *Plasmodium vivax* has become the dominant and more stable malaria parasite species [2, 3], as has been reported in other regions of the world where *P. vivax* and *Plasmodium falciparum* are sympatric [4–6]. Hence, a renewed and sustained effort will be required to eliminate *P. vivax*. A greater understanding of the antibody dynamics to *P. vivax* in this low-transmission region will be essential for developing and implementing key elimination tools, such as vaccines and improved surveillance methods [7].

Following *P. vivax* infections in Thai patients, IgG responses are induced to a number of different *P. vivax* antigens [8–11], despite the relatively low transmission of *P. vivax* in this region. Two studies provide evidence that IgG responses to both pre-erythrocytic and blood-stage *P. vivax* antigens can be maintained for at least 1 year in the absence of detectable blood-stage infections, suggesting acquisition of immunity [12, 13]. Whilst these previous studies have established that IgG responses do develop following *P. vivax* infections in Thailand, and that these responses can be long-lasting, there is still a paucity of information concerning IgG responses on a community-wide level encompassing all age groups in low transmission settings.

In this study, IgG levels were measured against several *P. vivax* antigens within a comprehensive group of volunteers, using plasma samples from a cross-sectional survey conducted in western Thailand in 2012. The proteins assessed included PVX_081550 (a putative StAR-related lipid transfer protein), the putative GPI-anchored micronemal antigen (GAMA), P12, P41, the asparagine-rich protein (ARP), five members of the reticulocyte binding protein (RBP) family and the circumsporozoite protein (CSP). PVX_081550, GAMA, P12, P41, and ARP are all potential blood-stage vaccine candidates based on their *P. falciparum* orthologs which are known or predicted to be involved in erythrocyte invasion [14–17]. The RBP family is thought to be responsible for the restricted host cell selectivity of *P. vivax*, making these antigens potential targets for a vaccine that interrupts blood-stage infections [18, 19]. CSP is a well-studied pre-erythrocytic candidate; the *P. falciparum* orthologue is the major component of the RTS,S vaccine [20]. The association of IgG levels with asymptomatic *P. vivax* infections, age, and spatial location were all explored, and how this information can contribute to the development of new or improved tools to facilitate elimination of *P. vivax* from western Thailand is discussed.

## Methods

### Cross-sectional survey

The cross-sectional survey was conducted in Kanchanaburi and Ratchaburi Provinces of western Thailand in September 2012 (Nguitragool et al. submitted). Briefly, 4309 volunteers were surveyed in eight villages, where *P. vivax* infection prevalence varied from 1.45 to 7.4%. Prevalence was defined by a positive quantitative PCR (known as qMAL), with species identification by single-plex qPCR, both as described [21, 22]. From all participants, 250 μl of capillary blood were collected by finger prick into an EDTA-containing microtainer. A 50 μl portion was immediately preserved for RNA extraction on-site, whilst the remaining blood was separated into pellet (for DNA extraction) and plasma on the same day and stored at −20 and −80 °C, respectively.

The ‘Bongti moo 3’ village was identified as having one of the higher rates of malaria prevalence amongst the eight villages surveyed (4.1%): thus samples collected from 546 volunteers living in this village were selected for IgG analysis in this study. Of those, 22 volunteers had confirmed *P. vivax* infections by qPCR at the time of sampling, as detailed in Table 1. All 22 infections were considered asymptomatic. We defined asymptomatic individuals as having no fever at the time of blood collection (<37.5 °C), in addition to no history of fever or any other malaria symptoms (feeling ‘unwell’) within the previous 2 days. Temperature was measured with an infrared thermometer prior to blood collection. As individuals were not seen again after this time, we do not know whether they subsequently developed febrile symptoms, a limitation of our definition of asymptomatic individuals. The feature of asymptomatic infections reflected the total cross-sectional survey, where 91.7% of *P. vivax* infections were classified as asymptomatic (Nguitragool et al. pers. Comm.). Age of the volunteers ranged from 6 months to 87 years, and 47% were male. Other demographic and epidemiological variables are shown in Table 1.

**Table 1:** Characteristics of the 546 cross-sectional survey volunteers

Variable	Value
*Plasmodium* infection, number (%)	35 (6.4%)
*P. vivax*	20
*P. falciparum*	5
Mixed *P. vivax/P. falciparum*	2
Undetermined	8
Age (years), median (range)^a^	22 (0.5–87)
0–6 years, number	89
7–12 years, number	107
13–17 years, number	48
18 years and older, number	298
Gender, number (%)^a^	
Male	254 (46.7%)
Female	289 (53.3%)
GPS location, number (%)	
Group 1 (close to Myanmar)	80 (14.7%)
Group 2 (close to local facilities)	466 (85.3%)
Length of time in Thailand, number (%)^a^	
More than 2 months	537 (100%)
Slept outside village last month, number (%)^a^	
Yes	2 (0.4%)
No	534 (99.6%)
Anti-malarial drugs taken in last 2 months, number (%)^a^	
Yes	4 (0.7%)
No	532 (99.3%)
Taking current medications, number (%)^a^	
Yes	5 (0.9%)
No	531 (99.1%)
Bed net used last night, number (%)^a^	
Yes	529 (98.7%)
No	7 (1.3%)
How long house has had bed net, number (%)^a^	
Past 6 months	66 (12.4%)
1 year or more	1 (0.2%)
2 years or more	464 (87.4%)
Feeling unwell today, number (%)^a^	
Yes	3 (0.6%)
No	533 (99.4%)
Fever last 2 days, number (%)^a^	
Yes	2 (0.4%)
No	534 (99.6%)

^a^Demographic/epidemiological details were not recorded for all 546 volunteers: numbers as shown

**Fig. 1:**
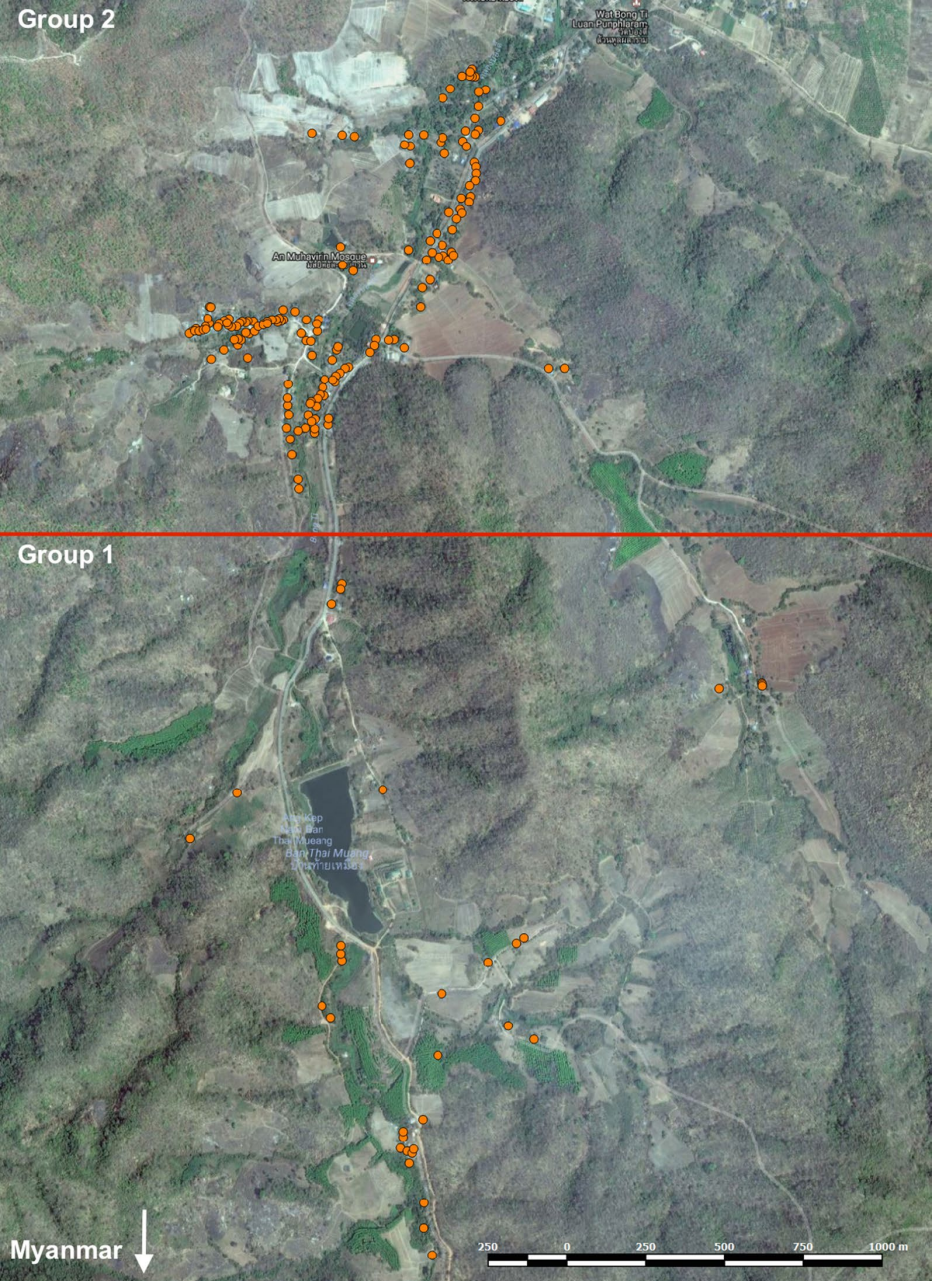
Location of houses of the study families in Bongti moo 3. Group 1 (n = 80) contains houses that lie closer to the border with Myanmar, and group 2 (n = 466) contains houses that are closer to the local health facilities and schools

The location of each volunteer’s home was recorded by GPS. Based on the distance from the Myanmar border, each volunteer was assigned to either group 1 (living close to the border) or 2 (away from the border and closer to the local school, malaria clinic, and other health facilities, Fig. 1).

### Protein selection and production

IgG responses were measured to 11 different *P. vivax* protein constructs: the full-length ectodomains of PVX_081550, GAMA (PVX_088910), P12 (PVX_113775), P41 (PVX_000995), and ARP (PVX_090210) [23]; CSP (based on a Korean isolate [24]); and five members of the RBP family (RBP1a, RBP2a, RBP2c, RBP2-P2, and RBP1b) [18]. For RBP2c, the fragment does not include the erythrocyte-binding domain, which encompasses residues 128–429, and is therefore referred to as RBP2cNB [25].

Proteins were expressed and purified using hexa-histidine tags in three collaborating laboratories as previously described, using HEK293E [26] or *Escherichia coli* [18, 19, 27] expression systems. PVX_081550, GAMA, P12, P41, and ARP also contained a Cd4 tag to assess protein expression levels [26].

### Antibody measurements

IgG levels were measured using a multiplexed bead-based assay as previously described [26]. Briefly, 2.5 × 10^6^ COOH microspheres (Luminex Corp) were incubated for 20 min at room temperature in 100 mM monobasic sodium phosphate (pH 6.2); 50 mg/ml sulfo-NHS (*N*-hydroxysulfosuccinimide sodium salt) was added to convert the protein’s carboxyl groups to amine-reactive NHS esters and 50 mg/ml of EDC [*N*-(3-dimethylaminopropyl)-*N*′-ethylcarbodiimide hydrochloride**]** used to cross-link *P. vivax* proteins to the microspheres, with incubation at 4 °C overnight. Optimal protein concentrations were determined experimentally to generate a log-linear standard curve with a positive control plasma pool prepared from immune Papua New Guinean (PNG) donors (see below). This enabled all plasma samples to be tested at one dilution with all proteins multiplexed.

50 μl of protein-conjugated microspheres in buffer were added to a 96-well V bottom tissue-culture plate (500 microspheres per well) and incubated with 50 μl of test plasma at a 1/100 dilution for 30 min at room temperature on a plate shaker. All dilutions were made in phosphate buffered saline containing 1% bovine serum albumin and 0.05% (v/v) Tween-20 (denoted as PBT), and all samples were run singularly. Following the incubation, the plate was centrifuged at 600×*g* for 3 min and the microspheres washed three times with 100 μl of PBT. The washed microspheres were incubated for 15 min with 1/100 detector antibody, PE-conjugated anti-human IgG Fc (1 mg/ml, Jackson ImmunoResearch), at room temperature on a plate shaker. The microspheres were then washed and assayed on a Bio-Plex 200^®^ and the results expressed as the median fluorescent intensity (MFI).

On each plate, a twofold serial dilution from 1/50 to 1/25,600 of a positive control plasma pool (generated from PNG adults) was included to generate a standard curve to allow standardization between plates. If the standard curve failed on a plate, the results for that protein were excluded. If less than 15 beads were counted for a bead region of a well, the result for that plasma sample was excluded.

### Statistical analysis

The raw MFI results were converted to relative antibody units using protein-specific standard curve data. A log–log model was used to obtain a more linear relationship, and a five-parameter logistic function was used to obtain an equivalent dilution value compared to the PNG control plasma [ranging from 1.95 × 10^−5^ (or 1/51,200) to 0.02 (or 1/50)] [26]. The interpolation was performed in R.

Five of the proteins contained Cd4 tags [26]. As such tags are known to induce an antibody response in some individuals [28], the measured antibody level (Ab_meas_) was assumed to be a combination of the true antibody response to the protein (Ab_true_) and the antibody response to the Cd4 tag (Ab_Cd4tag_): log(Ab_meas_) = log(Ab_true_) − βlog(Ab_Cd4tag_). The slope of the curve (β) was estimated using linear regression, and Ab_true_ was then estimated as the antibody response that would be measured when the Cd4 response is at the lowest measured value (1.95 × 10^−5^) [26].

Using the interpolated and Cd4-transformed data, further analysis and data presentation was performed in Prism version 6 (GraphPad, USA) or Stata version 12.1 (StataCorp, USA). Antibody values were log_10_-transformed and differences in IgG levels between categorical exposure variables determined using unpaired two-sample t tests or ANOVA with Sidak’s multiple-comparisons test. A multivariate linear regression model was used to determine the most significant associations with antibody level. The significance threshold was p = 0.05. Data are presented using box-plots (median), with error bars showing the 5–95 percentile and dot points the outliers. All antibody data generated (relative antibody units, not log_10_-transformed) are provided in Additional file 1, along with the appropriate epidemiological data.

### Mathematical methods

Cross-sectional data on age-specific seropositivity to *P. vivax* antigens are assumed to represent cumulative exposure of the population to *P. vivax*, allowing estimation of exposure patterns. Seropositivity was set at the mean plus two times the standard deviation of uninfected individuals aged 2–3 years from the study (young enough to have had limited exposure but old enough to no longer have maternally-acquired antibodies [13]). Two different models for describing seroprevalence curves were compared [29]. Model 1 assumes that the seroconversion rate is constant over time (i.e., exposure is constant over time). The seroconversion rate is λ and the seroreversion rate is ρ. The predicted proportion of individuals of age *a* that is seropositive is given by:

$$P\left( a \right) = \frac{\lambda }{\lambda + \rho }\left( {1 - e^{{ - \left( {\lambda + \rho } \right)a}} } \right)$$

In Model 2, the assumption was a stepwise reduction in transmission *t*_*c*_ years ago. Before reduction, it is assumed the seroconversion rate was *λ*_*0*_, and that it drops to *λ*_*c*_. The proportional reduction is denoted to be *γ* = *λ*_*c*_/*λ*_*0*_. Again, the seroreversion rate is ρ. The proportion of individuals of age *a* that is seropositive is given by the following formula from Yman et al. [29].

$$P\left( a \right) = \left\{ {\begin{array}{ll} {\frac{{\lambda_{c} }}{{\lambda_{c} + \rho }}\left( {1 - e^{{ - \left( {\lambda_{c} + \rho } \right)a}} } \right)} & \quad {a \le t_{c} } \\ {\frac{{\lambda_{c} }}{{\lambda_{c} + \rho }} + \frac{{\left( {\lambda_{0} - \lambda_{c} } \right)\rho }}{{\left( {\lambda_{0} + \rho } \right)\left( {\lambda_{c} + \rho } \right)}}e^{{ - \left( {\lambda_{c} + \rho } \right)t_{c} }} - \frac{{\lambda_{0} }}{{\left( {\lambda_{0} + \rho } \right)}}e^{{ - \left( {\lambda_{c} + \rho } \right)a}} e^{{ - \left( {\lambda_{0} - \lambda_{c} } \right)\left( {a - t_{c} } \right)}} } & \quad {a >t_{c} } \\ \end{array} } \right.$$

The likelihood that the model fits the data was calculated using a binomial distribution. The models were fitted in a Bayesian framework using Markov Chain Monte Carlo methods with uninformative uniform priors. Posterior median parameter estimates and 95% credible intervals are presented in Additional file 2.

## Results

### Multiple *Plasmodium vivax* proteins are immunogenic in western Thailand

**Fig. 2:**
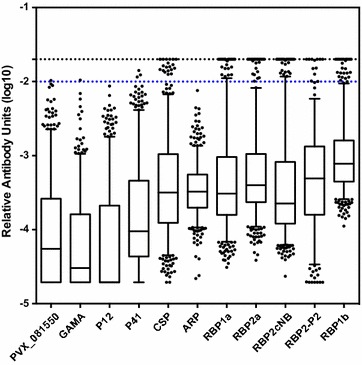
IgG levels to 11 *Plasmodium vivax* proteins in Thai volunteers. Relative antibody units, as compared to the immune control plasma pool, for each protein were calculated for everyone (n = 315–546). *Box plots* represent the median and interquartile range of log_10_-transformed data, *error bars* show the 5–95 percentile and *filled circles* show outlier values. The 1/50 and 1/100 values of the immune control plasma pool are shown in *black* and *blue lines*, respectively

IgG levels to the 11 *P. vivax* proteins were first analysed in all 546 samples collected in Bongti moo 3. Overall, IgG levels were lower in comparison to positive control immune plasma pooled from PNG adults (Fig. 2). Despite the overall lower levels, a proportion of volunteers had relatively high IgG levels, with some reaching (or exceeding) levels equivalent to a 1/50 dilution of the pooled plasma from PNG adults. Whilst the antibody levels cannot be directly compared between the different proteins, a higher proportion of volunteers reached the equivalent of a 1/100 dilution of the positive control plasma against the RBPs and CSP (3.5–5.7%), compared to the other four blood-stage proteins (0–0.6%). For these four blood-stage proteins, correction for the Cd4 tag will have resulted in slightly lower estimates of antibody units. IgG levels between the five RBPs tested were significantly correlated (pairwise correlation coefficients of 0.35–0.76, all p < 0.05 after Sidak’s multiple-comparisons correction).

### IgG levels are higher in individuals with a current *Plasmodium vivax* infection

**Fig. 3:**
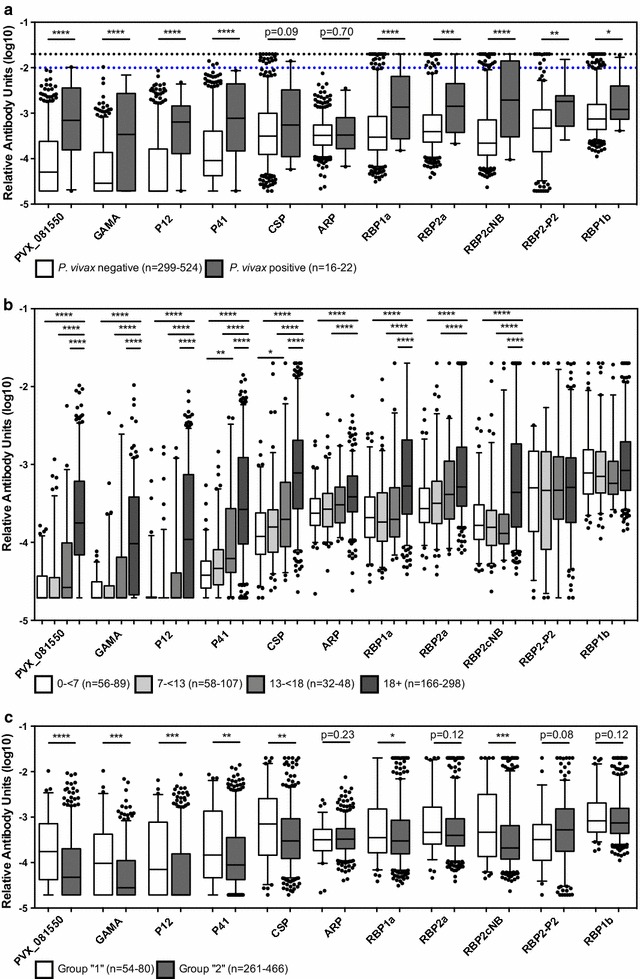
IgG levels to 11 *Plasmodium vivax* proteins in relation to other variables. **a** The Thai volunteers were divided into *P. vivax*-negative (n = 299–524) and *P. vivax*-positive (n = 16–22) to determine associations of IgG levels with current infection. Statistical difference between the two groups was assessed using the Student’s t test. The 1/50 and 1/100 values of the immune control plasma pool are shown in *black* and *blue lines*, respectively. **b** The volunteers were divided into four age groups, 0–6 years (n = 56–89), 7–12 years (n = 58–107), 13–17 years (n = 32–48) and 18 years and older (n = 166–298), to determine associations of IgG levels with age. Statistical difference between age groups was assessed using a one-way ANOVA with Sidak’s multiple-comparisons test. **c** The volunteers were divided into two spatial groups (see Fig. 1): those living near the border, group 1 (n = 54–80), and those living near health facilities, group 2 (n = 261–466), to determine associations of IgG levels with spatial heterogeneity. Statistical difference between the two groups was assessed using the Student’s t test. In all panels, *box-plots* represent the median and interquartile range, *error bars* show the 5–95 percentile and *filled circles* show outlier values. ****p < 0.0001, ***p < 0.001, **p < 0.01, *p < 0.05

IgG levels were next compared between individuals who had a current *P. vivax* infection and those who did not. Despite the fact that infected individuals were asymptomatic (and hence had a low parasite density, 0.125–444 copies *P. vivax* 18S gene/μl), they had significantly higher IgG levels to nine of 11 *P. vivax* proteins (t test, p = 0.018 to <0.0001) (Fig. 3a). One exception was the pre-erythrocytic antigen CSP; whilst there was a trend towards higher IgG levels in volunteers with a current infection, this did not reach statistical significance (p = 0.09). Even in the currently-uninfected volunteers, there was a proportion that had exceptionally high IgG levels against CSP detected, equal to or exceeding 1/100 and 1/50 dilutions of the immune pool from PNG. The other exception was for the blood-stage protein ARP. In this case, the IgG levels were remarkably similar between the two groups, with less variation in levels compared to the other proteins (Fig. 3a). As seen for CSP, in the group of *P. vivax*-uninfected volunteers there were some exceptionally high IgG levels detected against the RBP proteins, again equaling or exceeding that observed in the immune PNG control pool.

### IgG levels increase with age

The association of IgG level with age was next assessed. For 33 volunteers the age was recorded as a bracket (i.e., 0–6 years, 7–12 years, 13–17 years, 18 years and older), and hence association of antibodies with age was conducted by comparing these defined age groups. For nine of the 11 *P. vivax* proteins, IgG levels were significantly higher in adults aged 18 years and older compared to those in the two groups of younger children (0–6 years and 7–12 years, p < 0.0001) (Fig. 3b). For multiple proteins this difference was also evident compared to older children aged 13–17 years (PVX_081550, GAMA, P12, P41, CSP, RBP1a, and RBP2cNB, p < 0.0001). For P41 and CSP, there was also a significant difference between young children 0–6 years and older children aged 13–17 years (p = 0.003 and p = 0.018, respectively). For these nine antigens where an association with age was clear, there were no exceptionally high IgG levels detected in the youngest age group of children. For RBP2-P2 and RBP1b, there was no significant association with age (Fig. 3b). For these two proteins, IgG levels were very similar between the four defined age groups, and in addition high IgG levels were detected in even the youngest group of children aged 0–6 years. For the 509 volunteers where an exact age was recorded, there was a statistically significant correlation between age and IgG level to the same nine of 11 proteins (Spearman r correlation coefficients of 0.25–0.65, n = 389–509). There was a weak but significant correlation for RBP1b (r = 0.1, p = 0.02, n = 509).

**Fig. 4:**
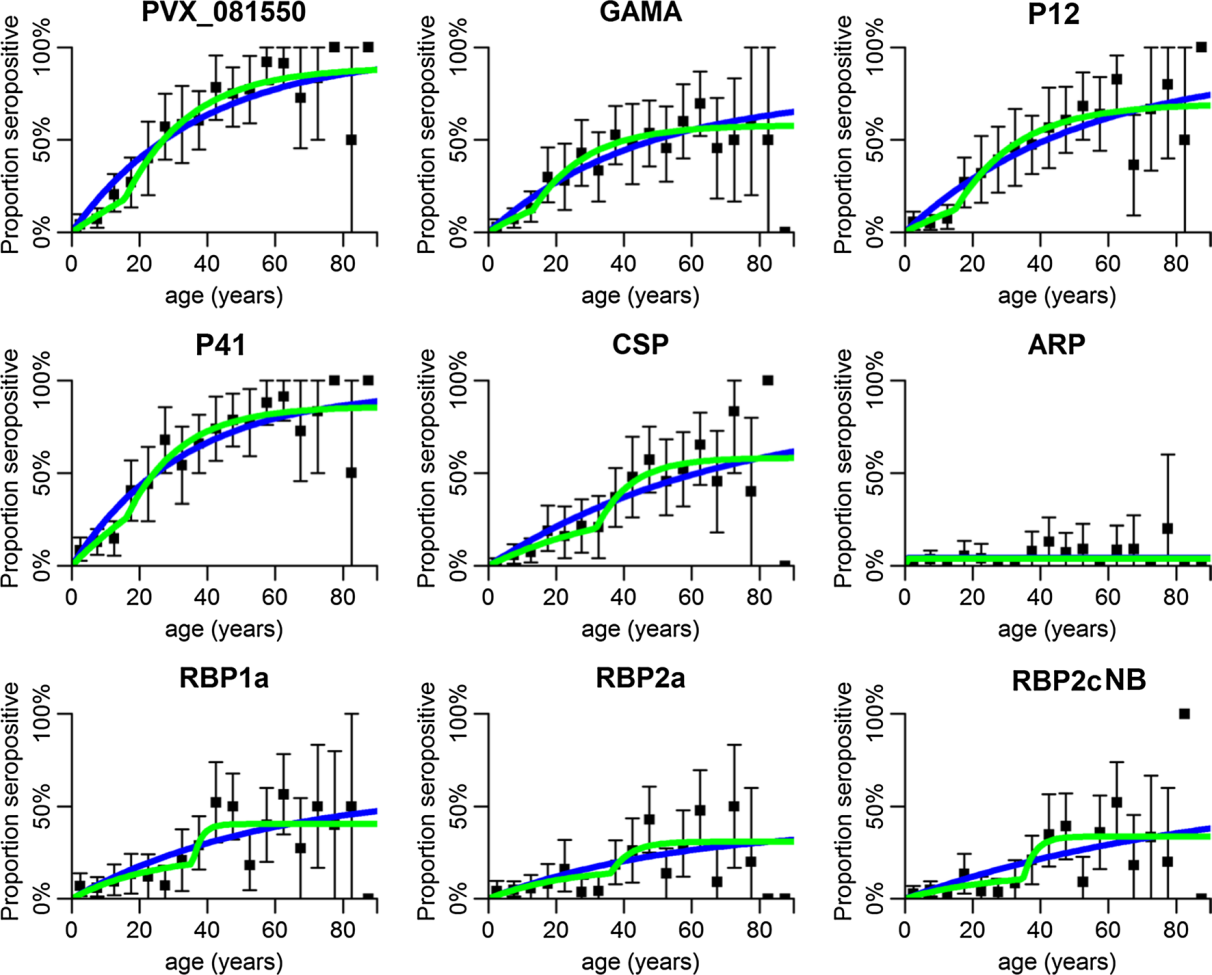
Serocatalytic models fitted to cross-sectional data on age-dependent seropositivity for nine *Plasmodium vivax* antigens. *Black squares* denote the proportion of seropositive individuals and *vertical bars* denote the 95% confidence interval. Model 1 (*blue*) assumes a constant seroconversion rate over time. Model 2 (*green*) assumes a stepwise reduction in seroconversion rate

Age-dependent trends in the proportion of seropositive individuals were also analysed using reversible serocatalytic models [29, 30], for the nine proteins where an association with age was evident. There was a step change in the seropositive proportion between the ages of 15 and 30 (Fig. 4), with Model 2 providing the better fit for most proteins. This suggests either a reduction in transmission 15–30 years ago, or an age-dependent change in exposure patterns, for example due to increased exposure to mosquito bites during the ages of 15–30 years due to changing work patterns. The results from the serocatalytic models also indicate long-lived IgG responses, with half-lives of more than 20 years for most antigens (Additional file 2).

### IgG levels can reflect spatial heterogeneity

Given the interest in using antibody levels for surveillance [31], spatial heterogeneity in IgG levels was assessed. As volunteers all came from one village and lived within 3 km of each other, a crude distinction was made based on ease of reaching the volunteer’s home. Group 1 volunteers lived closer to the Myanmar border and further from local health facilities, whilst group 2 volunteers lived further from the border and closer to these facilities (Fig. 1). Significant levels of spatial heterogeneity were observed in IgG levels to seven of the 11 *P. vivax* protein constructs tested (Fig. 3c); IgG levels to PVX_081550, GAMA, P12, P41, CSP, RBP1a, and RBP2cNB were significantly higher in group 1 than in group 2 (t test, p = 0.047 to < 0.0001). There was no significant difference in IgG level between the two spatial groups for ARP, RBP2a, RBP2-P2 and RBP1b, which includes the antigens for which there was no age association, suggesting that exposure may have limited effect on immune responses to these antigens.

Other demographic and epidemiological factors were also explored in relation to the antibody response, where there were a significant number of people in two or more different groups (see Table 1). For most *P. vivax* proteins there was no statistically significant difference in IgG level between males and females, except for RBP1a where males had slightly higher IgG levels (n = 542, p = 0.028, t test). An association was also evident for RBP1a with bed net ownership; IgG levels were significantly higher in individuals who had owned a bed net only for the past 6 months, compared to those who had owned one for more than 2 years (n = 530, p = 0.03). No association with bed net ownership was observed for the other ten proteins, suggesting the association between anti-RBP1a antibodies and bed net ownership may have been a chance occurrence.

### Age and current *Plasmodium vivax* infection are the best predictors of IgG level

Finally, the best predictors of antibody level were explored using a multivariate linear regression model. For each protein, any variables that had a significant association with antibody level were initially included in the model. A backward, stepwise, elimination process was used to determine the final best-fit model, with variables that were not significant when adjusted removed (Table 2). Age was assessed as both a linear and quadratic term, with the assumption that antibody levels first increase with age but that this reaches a plateau. For most proteins where there was a significant association with age, this was true for both the linear and quadratic terms in the final model. For nearly all proteins, age and current *P. vivax* infection remained significantly associated with antibody level. For CSP, only age remained significantly associated (spatial location was univariately associated). For GAMA, only age and spatial location remained significantly associated. For ARP, only age was significantly associated univariately, and for RBP2-P2 only current *P. vivax* infection was significantly associated univariately. For RBP1a, sex and bed net usage, in addition to age and current *P. vivax* infection, remained significantly associated. For PVX_081550, P12, and RBP2cNB, spatial location, in addition to age and current *P. vivax* infection, remained significantly associated.

**Table 2:** Multivariate linear regression model: key variables associated with antibody level

	PVX_081550	GAMA	P12	P41	CSP	ARP	RBP1a	RBP2a	RBP2cNB	RBP2-P2	RBP1b
Age, linear											
n	506	389	506	504	509	506	495	506	509		
Coefficient	0.32***	0.31***	0.17***	0.37***	0.28***	0.13***	0.13***	0.088***	0.13***		
95% CI	0.25, 0.40	0.22, 0.4	0.15, 0.2	0.29, 0.45	0.2, 0.35	0.081, 0.18	0.11, 0.16	0.066, 0.11	0.11, 0.15		
Age, quadratic											
n	506	389		504	509	506					509
Coefficient	−0.019**	−0.025***		−0.025***	−0.016**	−0.015***					0.0035**
95% CI	−0.03, −0.0079	−0.038, −0.011		−0.038, −0.013	−0.026, −0.0053	−0.022, −0.007					0.001, 0.006
Current *P. vivax* infection											
n	506		506	504			495	506	509	315	509
Coefficient	0.66***		0.69***	0.51**			0.42**	0.34**	0.58**	0.55***	0.25*
95% CI	0.34, 0.97		0.36, 1.021	0.18, 0.84			0.11, 0.73	0.091, 0.59	0.23, 0.93	0.27, 0.82	0.044, 0.46
GPS location											
n	506	389	506						509		
Coefficient	−0.26**	−0.26**	−0.22*						−0.24*		
95% CI	−0.42, −0.1	−0.45, −0.068	−0.42, −0.026						−0.43, −0.045		
Sex											
n							495				
Coefficient							−0.14**				
95% CI							−0.24, −0.036				
Bed net usage											
n							495				
Coefficient							−0.2*				
95% CI							−0.37, −0.029				

Only data are for variables that were included in the final model are shown. * p < 0.05, ** p < 0.01 and *** p < 0.001. Note that ages were divided by 10 for this model, so coefficients represent 10-year increases in age

## Discussion

In this study, it was observed that IgG antibodies to multiple *P. vivax* proteins are acquired in individuals living in an endemic region of western Thailand, despite the overall low transmission. The level of IgG observed varied greatly amongst volunteers of this cross-sectional survey. In some individuals, IgG levels were equivalent to the immune control plasma pool made from PNG adults, whilst in others no protein-specific IgG was detected. Ten of 11 *P. vivax* proteins tested are expressed during the blood-stage of the parasite’s lifecycle, and it is likely their proposed location on the surface of merozoites makes them immunogenic targets [14, 32, 33]. PVX_081550 is an exception, as the *P. falciparum* orthologue of this protein locates to the parasitophorous vacuole, although there is some evidence it may be transferred into the apical organelles of merozoites [34]. The CSP protein locates to the surface of the sporozoite, and is well described as being immunogenic [34]. Recent results using samples from the same region of western Thailand demonstrated that RBP1b was poorly immunogenic in symptomatic *P. vivax* patients [18]. The current results show that this protein is immunogenic in asymptomatic *P. vivax* patients, and hence further research should investigate a potential link between IgG levels to RBP1b and association with protection from clinical malaria in this region.

Of the 546 volunteers included in this study, the majority was not infected with *P. vivax* (nor any other *Plasmodium* spp.) during the time of blood collection. Despite this, and considering the overall low transmission in the region, many volunteers had detectable IgG levels. The results from the serocatalytic model are consistent with IgG antibody responses being relatively long lived. Whilst this is likely the case, as the data were from a cross-sectional survey the relative longevity of the observed IgG antibodies cannot be concluded. Further studies utilizing samples from longitudinal surveys will be required, as has recently been done for CSP [13]. As these previous results indicated CSP is able to induce long-lived IgG responses in a similar population in western Thailand [13], this may account for the lack of a statistically significant increase in IgG level detected in volunteers with a current *P. vivax* infection. In addition, the pre-erythrocytic stage expression of this protein likely also contributes, as only new (and not relapsing) infections would likely generate new exposure to this antigen.

A higher IgG level was observed to nine of ten blood-stage proteins in volunteers with a current *P. vivax* infection at the time of sampling. This difference was statistically significant despite the low number of infected volunteers (n = 22) and the low antigenic input of these asymptomatic infections. The greatest differences in mean IgG levels between individuals currently uninfected and those infected with *P. vivax* were observed for the proteins PVX_081550, P41, and RBP2cNB. Antibodies to both PVX_081550, or StAR-related lipid transfer protein, and P41 have recently been associated with protection from clinical malaria in PNG children [26]. RBP2cNB has also been associated with protection from clinical disease in PNG children; however, after accounting for the fact that antibodies to the various RBPs are co-acquired and correlated, only RBP1a and RBP2b remained significantly associated [25]. IgG levels to RBP2b were not assessed in the current study. Whilst the results suggest that current asymptomatic *P. vivax* infections induce a boosting of the IgG response, at least at a community-wide level, the higher IgG levels could also reflect a higher risk of infection (and hence higher lifetime exposure) in these volunteers. Further analysis using samples from longitudinal cohorts, including volunteers with symptomatic infections, will be required to determine whether these IgG antibodies are indeed associated with exposure or protection from clinical disease. Parasite clearance times were not determined for infected individuals in this cross-sectional survey.

As expected [35–39], IgG levels to most of the proteins were higher in adults (18 years and older) compared to children. The serocatalytic models indicated that either there was a reduction in transmission 15–30 years ago, or that during the ages 15–30 a difference in risk of exposure exists. As mosquitoes in Thailand are known to bite outdoors, this fits with an increased risk in exposure to *P. vivax* in this age group who is likely working outdoors [3]. Two exceptions were found: IgG levels to both RBP2-P2 and RBP1b were no different between the four defined age categories, nor did they correlate with age. Previous research also identified no association of IgG levels to RBP2-P2 with age in PNG [25], although this was not the case for RBP1b. This suggests that in western Thailand IgG antibodies to these two RBPs could be acquired early in life, although this would be unexpected given the low transmission in this region. However, antibody responses to these two RBPs could be long lasting even in the absence of new infections. An alternative explanation is the existence of cross-reactive antibodies in this region to these protein constructs, which could generate a high level of non-specific antibodies. Using a multivariate linear regression model, the association with age reached a plateau in adults for most proteins. Age and current *P. vivax* infection status had the most significant influence on antibody levels in this study.

The use of antibody responses for identifying regions or hot spots of higher malaria transmission amongst a wider area of low transmission is gaining increasing interest [40]. All volunteers in this study lived within a limited area (3 km); however, spatial heterogeneity in the IgG levels to a subset of the *P. vivax* proteins was still identified. For those proteins, IgG levels were significantly higher in individuals living closer to the Myanmar border and further away from local health care facilities. Whilst travel to Myanmar is a known risk factor for malaria parasite infections in Thailand (Nguitragool et al. submitted), the volunteers in this village did not report sleeping outside their local village within the last month, and all had been living in Thailand for more than 2 months. Further details on their travel history within the past few years would be required to determine if they had been exposed to a greater risk of infection compared to other volunteers in the study. Overall, the results do support the use of antibody responses as tools to identify regions of ongoing malaria transmission: asymptomatic infections are associated with increased IgG levels, and even a crude spatial separation can elicit a significant difference in this level. However, it is important to note that the impact of spatial location on IgG level was not as crucial as the influence of age and current *P. vivax* infections in this population, with most associations with spatial location lost in the multivariate model.

## Conclusions

Here, IgG levels to 11 *P. vivax* proteins in over 500 individuals are reported. It is demonstrated that all proteins are recognized by a proportion of the volunteers, even in young children and those uninfected with *P. vivax* parasites. These proteins are also recognized in other endemic regions, such as Cambodia [23] and the Solomon Islands [26], and hence these responses are not specific to western Thailand. Asymptomatic *P. vivax* infections were associated with higher IgG levels to most proteins, despite the low density of such infections. Increased IgG levels were also associated with an increase in age for most proteins. Further investigation of such antibody levels in low-transmission regions, such as Thailand, has the potential to provide valuable information for the development and implementation of elimination tools such as surveillance markers and vaccines.

## Additional files

**Additional file 1.**All antibody data generated, and epidemiological data analysed, for the current study. Antibody data is given in Relative Antibody Units (not log transformed).

**Additional file 2.** Parameter estimates for serocatalytic models. Parameters are presented as posterior medians with 95% credible intervals. All parameters had improper uniform prior distributions.

## Abbreviations

GAMA: GPI-anchored micronemal antigen
ARP: asparagine-rich protein
RBP: reticulocyte binding protein
CSP: circumsporozoite protein
RBP2cNB: RBP2c non-binding region
sulfo-NHS: *N*-hydroxysulfosuccinimide sodium salt
EDC: *N*-(3-dimethylaminopropyl)-*N*′-ethylcarbodiimide hydrochloride
PNG: Papua New Guinean
PBT: phosphate buffered saline containing 1% bovine serum albumin and 0.05% (v/v) Tween-20
MFI: median fluorescent intensity
qPCR: quantitative PCR
