# Supplementary material for: Asymptomatic Plasmodium vivax infections induce robust IgG responses to multiple blood-stage proteins in a low-transmission region of western Thailand
Source: Malar J. 2017 Apr 28;16:178. doi: 10.1186/s12936-017-1826-8 (PMC5410030; doi:10.1186/s12936-017-1826-8)
Supplement: Supplementary file 2 — Additional file 2. Parameter estimates for serocatalytic models. Parameters are presented as posterior medians with 95% credible intervals. All parameters had improper uniform prior distributions. [file 12936_2017_1826_MOESM2_ESM.docx]

Additional File 2: Parameter estimates for serocatalytic models. Parameters are presented as posterior medians with 95% credible intervals. All parameters had improper uniform prior distributions.

|  | **Model 1 (constant exposure)** | | | **Model 2 (stepwise reduction in exposure)** | | | | |
| --- | --- | --- | --- | --- | --- | --- | --- | --- |
|  | ***λ*** | ***ρ*** | ***t*_half_ (years)** | ***λ_0_*** | ***γ*** | ***ρ*** | ***t_c_*** | ***t*_half_ (years)** |
| PVX_081550 | 0.026  (0.022, 0.030) | 0.0009  (0.0, 0.004) | 770  (173, ∞) | 0.050  (0.032, 0.198) | 0.26  (0.09, 0.48) | 0.004  (0.0002, 0.011) | 15.9  (7.5, 35.5) | 173  (63, 3465) |
| GAMA | 0.017  (0.013, 0.022) | 0.0052  (0.003, 0.0161) | 133  (43, 231) | 0.046  (0.017, 0.251) | 0.23  (0.06, 0.83) | 0.021  (0.002, 0.043) | 13.4  (3.4, 47.9) | 33  (16, 347) |
| Pv12 | 0.017  (0.015, 0.021) | 0.0019  (0.0, 0.0087) | 365  (80, ∞) | 0.043  (0.022, 0.149) | 0.22  (0.07, 0.59) | 0.011  (0.001, 0.026) | 15.3  (9.0, 32.2) | 63  (27, 693) |
| Pv41 | 0.028  (0.024, 0.034) | 0.0013  (0.0, 0.0059) | 533  (117, ∞) | 0.056  (0.033, 0.168) | 0.35  (0.13, 0.68) | 0.006  (0.001, 0.015) | 15.8  (8.8, 41.7) | 116  (46, 693) |
| CSP | 0.012  (0.001, 0.015) | 0.0020  (0.0, 0.0088) | 347  (79, ∞) | 0.063  (0.017, 1.257) | 0.14  (0.01, 0.45) | 0.014  (0.001, 0.027) | 31.3  (13.7, 41.8) | 50  (26, 693) |
| ARP | 0.294  (0.137, 0.528) | 6.28  (3.54, 9.82) | 0.11  (0.07, 0.19) | 1.628  (0.092, 9.069) | 0.16  (0.01, 0.86) | 6.66  (0.70, 9.80) | 28.8  (1.8, 58.5) | 0.10  (0.07, 0.99) |
| RBP1a | 0.011  (0.008, 0.015) | 0.0072  (0.0003, 0.0246) | 96  (28, 2310) | 0.562  (0.052, 1.649) | 0.02  (0.01, 0.19) | 0.031  (0.021, 0.045) | 35.9  (26.4, 40.9) | 22  (15, 33) |
| RBP2a | 0.007  (0.005, 0.013) | 0.0118  (0.0005, 0.0448) | 59  (15, 1386) | 0.415  (0.043, 1.366) | 0.02  (0.00, 0.16) | 0.039  (0.022, 0.068) | 36.3  (27.8, 49.4) | 18  (10, 32) |
| RBP2cNB | 0.007  (0.005, 0.010) | 0.0044  (0.0001, 0.0209) | 157  (33, 6931) | 0.682  (0.026, 1.559) | 0.01  (0.00, 0.18) | 0.035  (0.015, 0.048) | 35.8  (27.4, 42.5) | 20  (14, 46) |

A note on statistical inference of seroreversion rates from cross-sectional serological data. With data from a single cross-section it is usually not possible to differentiate between the case where seroreversion occurs (*ρ* > 0) and the case where seroreversion does not occur (*ρ* = 0). This results in large estimates for the half-life of seroreversion with wide credible intervals. The wide credible intervals reflect the limited statistical power to estimate the seroreversion rate from this data. More accurate estimation of the seroreversion would require longitudinal data.
